# Using activity and sociability to characterize collective motion

**DOI:** 10.1098/rstb.2017.0015

**Published:** 2018-03-26

**Authors:** David J. T. Sumpter, Alex Szorkovszky, Alexander Kotrschal, Niclas Kolm, James E. Herbert-Read

**Affiliations:** 1 Mathematics Department, Uppsala University, Uppsala, Sweden; 2 Zoology Department, Stockholm University, Stockholm, Sweden

**Keywords:** collective behaviour, factor analysis, fish, *Poecilia reticulata*, personality

## Abstract

A wide range of measurements can be made on the collective motion of groups, and the movement of individuals within them. These include, but are not limited to: group size, polarization, speed, turning speed, speed or directional correlations, and distances to near neighbours. From an ecological and evolutionary perspective, we would like to know which of these measurements capture biologically meaningful aspects of an animal's behaviour and contribute to its survival chances. Previous simulation studies have emphasized two main factors shaping individuals' behaviour in groups; attraction and alignment. Alignment responses appear to be important in transferring information between group members and providing synergistic benefits to group members. Likewise, attraction to conspecifics is thought to provide benefits through, for example, selfish herding. Here, we use a factor analysis on a wide range of simple measurements to identify two main axes of collective motion in guppies (*Poecilia reticulata*): (i) sociability, which corresponds to attraction (and to a lesser degree alignment) to neighbours, and (ii) activity, which combines alignment with directed movement. We show that for guppies, predation in a natural environment produces higher degrees of sociability and (in females) lower degrees of activity, while female guppies sorted for higher degrees of collective alignment have higher degrees of both sociability and activity. We suggest that the activity and sociability axes provide a useful framework for measuring the behaviour of animals in groups, allowing the comparison of individual and collective behaviours within and between species.

This article is part of the theme issue ‘Collective movement ecology’.

## 1. Introduction

One of the key questions in the study of collective animal behaviour is how the environment, through natural selection, shapes the behaviour of individuals that live in groups [1–6]. Many of the examples of cooperation studied in this Special Issue involve interactions between individuals with low levels of relatedness (e.g. Sasaki *et al.* [7], Strandburg-Peshkin *et al*. [8], del Mar Delgado *et al.* [9], this issue). There are many other examples of such cooperation, including penguins huddling to exchange warmth [10], collective foraging and vigilance by sparrows [11], the coordinated escape waves of fish schools under predatory attack [12] and herds of mammals finding safety in numbers [13]. Within these groups, membership changes so rapidly that neither relatedness nor reciprocity can fully explain cooperation [14]. Equally, models of cooperation in which individuals interact with neighbours in static networks (e.g. [15]) do not capture the ever-changing interactions within fish schools, mammal herds, bird flocks and other groups.

The failure of relatedness, reciprocity or graph theory to explain cooperation in some animal groups makes the question of its evolution even more intriguing. Empirical and modelling studies have revealed the importance of two evolutionary explanations in particular: information transfer and dilution effects. In moving animal groups, information transfer occurs, for example, when one individual responds to the detection of a predator, and its neighbours respond in turn to the first individual's change in behaviour [12,16–19]. Then the neighbours of those neighbours respond, creating a wave of information that passes through the group [20,21]. This spread of information depends on individuals actively monitoring their neighbours and copying the decisions others may make. The continual exchange of mutually beneficial information can provide an advantage to all group members, and is unlikely to be exploited by cheats, because all individuals benefit from information exchange [12]. The resulting strategy is evolutionarily stable, in the sense that if one individual does not pass on information, both it and others suffer a cost [2]. The other evolutionary explanation, the dilution effect, is seen when individuals aggregate because they are less likely to become the selected prey item in the event of a predator attack [22]. Leaving the group incurs considerable costs, due to predators targeting, or being more successful at hunting, individuals in smaller groups [23].

A valid criticism of many of the early studies of collective animal behaviour was that they were limited to a description of group-level behaviour, while natural selection acts at the level of the individual. It was initially unclear from looking at the group how abstract ideas, such as ‘information spread’ or ‘dilution’, could be narrowed down to the behavioural responses of individuals to each other. Several computer models established the importance of repulsion, attraction and alignment responses in allowing rapid information transfer in moving animal groups [24–26]. These simple interactions could produce complex group-level responses, such as waves of turning passing through the group. Modelling studies showed that both information transfer, where individuals changed direction to collectively escape predators, and dilution effects, where individuals aggregated to mitigate individual risk, were evolutionarily stable outcomes [27]. These simulation results were supported by an experiment in which a predator that interacted with virtual prey would target prey that had weaker alignment or attraction rules, and had therefore become separated from the group [28].

With the importance of alignment and attraction established through modelling, the next experimental step was to identify these rules in an experimental setting [29]. Advances in tracking technology have allowed collective motion to be studied in great detail, identifying specific behavioural rules of interaction [30–32] while also quantifying the distances, bearing angles and relative orientations of near neighbours to describe the spatial structure of groups [3]. Indeed, the interplay between the positioning behaviour of individuals in groups, and the interactions that produce particular spatial configurations have been argued to be functionally equivalent [33]. These empirical studies showed that animal interactions were more complicated than simple alignment and attraction rules. Individuals’ interaction rules, focused around changes in speed and orientation [30,31] and intermittent locomotion [34], were affected by differences such as body size [35], and depended on whether individuals more often led or followed other individuals [36]. They were also dependent on the species in question [3], making it difficult to give broad categories by which to discuss within and between species differences about how individuals interact in moving animal groups. A further limitation of ‘rules of interaction’ studies is that they are not really about individuals *per se* [3]. With the exception of some studies [36,37], most have attempted to capture the average behaviour of how individuals interact when in groups, without regard to variation between individuals' behaviour.

In contrast with the averaging procedure of most collective motion studies, when studying differences between individual animals—in, for example, personality studies [38,39]—a useful approach has been to perform many different behavioural measurements and use ordination techniques such as principal components analysis (PCA) or factor analysis to reduce the data to a small number of explanatory variables [40]. Such an approach could also address some of the challenges in understanding collective motion, where there are many different measurements of group level properties, and where it is often unclear which of these measurements best capture the behaviour in which individuals tend to vary. Ordination techniques deal well with problems where the measurable properties such as speed, inter-individual distances and alignment are highly related to each other, as well as to the structure and size of the environment.

In this article, we infer inter-individual differences in how individuals interact by subjecting typical measurements of collective motion to a factor analysis. We first measure the properties of individuals in animal groups and use factor analysis to find the key behavioural components. By comparing groups from different experimental treatments, this approach allows us to shed some light on the key factors that are important in shaping collective motion. We first show that there is substantial variation in collective motion between different groups of guppies (*Poecilia reticulata*). This variation can be broadly described as occurring along two dimensions of collective ‘activity’ and ‘sociability’, which we find using factor analysis on a range of collective motion measurements. We then examine how populations of guppies that experience different levels of predation risk differ in these activity and sociability axes. We also describe how a process of sorting guppies based on their group level properties (average directional alignment) rapidly produces differences in sociability and activity between groups. We finally discuss how we might infer the personality of individuals living in groups from these different activity/sociability axes, and discuss how natural selection could shape such personality differences.

## 2. Experimental methods and measurements

### (a) Data

In this paper, we use data from three separate experiments we conducted, all involving open-field assays of single-sex groups of adult guppies (*P. reticulata*). In the first experiment, which we will refer to as the selection experiment [41], we looked for social differences between three breeding lines up-selected for brain size (i.e. large brain) and three lines down-selected for brain size (i.e. small brained) [42], testing pairs and groups of eight. Several aspects of collective motion, including all measurements used in this paper, were tested using linear mixed-effect models, and none were found to be significantly influenced by brain size [41]. However, this large dataset serves as a useful reference to attempt to partition the collective motion of guppy shoals into distinct behavioural axes. Because we found no differences in the shoaling behaviour of these lines, we combine all data of groups of eight from large- and small-brained lines together for this analysis (*n* = 29 groups of eight females, *n* = 28 groups of eight males). Here we use a unique approach to analyse the variation in collective motion between individuals and groups using factor analysis.

The first selection dataset is used primarily to establish the important factors in the collective movement of guppy shoals. To ensure independence, we then use the factors established in this first experiment to analyse differences between groups in the second and third experiments. We note, however, that the factors identified from the selection experiment were qualitatively similar to the ones identified in the other datasets (either separate or combined) if the factor analysis was run on these datasets separately (see the electronic supplementary material).

The second, predation experiment was designed to compare the shoaling behaviour of wild guppies from upstream and downstream populations of four rivers in Trinidad [43] (*n* = 78 groups of eight females, *n* = 51 groups of eight males). The downstream populations were subject to higher predation levels, and as such are known to have higher tendency to aggregate [44]. The third set of experiments involved *sorting* groups of female guppies from a laboratory population into groups that had relatively high to low average directional alignment [45] (*n* = 48 groups of eight females, *n* = 48 groups of eight males, mixed over 12 rounds).

For details of the individual experiments we refer the reader to their respective sections below, and to the individual articles [41,43,45], but we note that each of the experiments involved a similar set-up and the same analysis methods. All assays described in this paper were open-field tests with white arenas filled to depths of approximately 3–4.5 cm, into which groups of guppies (*n* = 8 fish) of the same sex and unfamiliar with the arena were placed. The predation experiment data were taken in a rectangular arena (1000 × 900 mm), where the guppies were remotely released from a holding container in the corner of the arena after an acclimation period of 5 min. The selection experiment and sorting experiment took place in circular arenas of 550 mm in diameter, and the guppies were manually released from the centre of the arena after 2 min of acclimation. All groups were filmed from above for at least 10 min at 24–25 frames per second, and fish's movements were tracked using semi-automated tracking software [46–48].

### (b) Measurements of collective motion

From each of these experiments, each tracked individual *i* is given coordinates *x*_*i*_(*t*) and *y*_*i*_(*t*) at each frame *t*. The change in position at each frame is then used to calculate the instantaneous speed *s*_*i*_(*t*) and headings *θ*_*i*_(*t*). These time series were then processed to calculate the following eight behavioural measures for each individual. The subscript *t* denotes an average over all frames. The form of data reduction (median, mean and maximum) was chosen so that the measures were as close as possible to normally distributed.

The speed of individual *i* is quantified by the median speed over all frames.
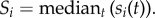


The turning rate for an individual is calculated using the absolute change in heading *θ*:
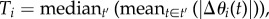
where *t*′ denotes any 1 s period with a mean speed over 1.5 mms^−1^. This type of measure can be used to quantify exploratory as opposed to goal-directed behaviour in fish [49].

The distance from the centre of the arena is calculated as the mean over all frames
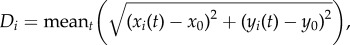
where *x*_0_ and *y*_0_ denote the coordinates of the arena centre. In open fields, larger distance from the centre often indicates reduced boldness in individual fish [50,51].

Cross-correlations in speed are used to quantify the tendency to synchronize with conspecifics. A pair of fish with a strong leader–follower relationship and a typical delay of *τ*_0_ between their movements will have a cross-correlation peak at |*τ*| ≈ *τ*_0_. Hence for individual *i* we use the maximum correlation peak over conspecifics *j*, defined as
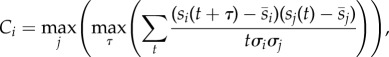
where *τ* is varied between −1 and 1 s, and 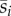 and *σ*_*i*_ represent the means and standard deviations, respectively, of *s*_*i*_(*t*).

The reaction time is also based on cross-correlations in speed. We use the average positive lag *τ* of a correlation peak
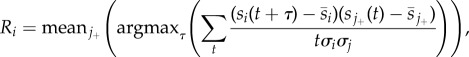
where *τ* is varied between −1 and 1 s and *j* + denotes all conspecifics where the maximum correlation is at *τ* > 0. We let *R*_*i*_ = 0 if individual *i* is leading all conspecifics (i.e. if all cross-correlation peaks are at negative *τ*).

The nearest neighbour alignment is given by
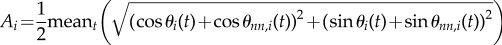
and the nearest neighbour distance is given by
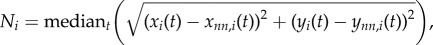
where *x*_*nn*,*i*_(*t*), *y*_*nn*,*i*_(*t*) and *θ*_*nn*,*i*_(*t*) denote the position and heading of the nearest neighbour with respect to individual *i* in frame *t*. The latter is a common measure of short-range aggregation [30,31].

Finally, the mean group size experienced, measuring long-range aggregation, is calculated by
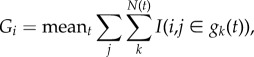
where at each frame *t* there are *N*(*t*) subgroups given by *g*_*k*_(*t*) for *k* ∈ {1, …, *N*(*t*)}. At each point in time, a fish is identified as belonging to a given subgroup if it is separated by less than 100 mm from at least one member of the subgroup. These subgroups are equivalent to connected network components if a network edge exists for all pairs of conspecifics separated by less than 100 mm. This distance was chosen to lie outside the typical interaction range of pairs of guppies [43], and close to the conventional four body lengths used for shoal membership [52]. The eight measurements we use are summarized in table 1. While most of these are general collective motion measurements, two (namely the speed correlation and speed delay) are specifically suited to the burst-and-glide movement of guppies, which is typical of how these fish swim.

**Table 1.** Measurements calculated from the trajectory data and used as input measures for the factor analysis.

measure	description
speed *S*_*i*_	characteristic speed
turning rate *T*_*i*_	turning angle per second
dist. from centre *D*_*i*_	distance from centre of arena
speed corr. *C*_*i*_	synchronization with conspecifics
reaction time *R*_*i*_	reaction time to conspecifics
n.n. align. *A*_*i*_	alignment with nearest neighbour
n.n. dist. *N*_*i*_	distance to nearest neighbour
group size *G*_*i*_	average group size experienced

The eight measures per individual in the selection experiment are used for factor analysis, as shown in the following section. For testing effects on these factors (such as predation, sorting and time), we used group means to avoid pseudo-replication.

## 3. Establishing the importance of sociability and activity

To establish the relationship between different measurements of collective motion, we used the dataset from the selection experiment. To reduce the multidimensionality of these data, we use exploratory factor analysis [53]. Factor analysis is related to, but also stands in contrast with PCA, which has previously been used in personality studies [40]. The key idea in PCA is to find the linear combinations of variables, known as components, which remove all correlation from the data, i.e. the eigenvectors of the correlation matrix. In PCA, there are as many principal components as there are input variables, ordered by how much variance they explain. In factor analysis, the idea is to reduce the data to a description in terms of a pre-specified number of components, *m*, that sufficiently capture the underlying covariances.

We performed factor analysis separately on all of the males and all of the females in the selection experiment. The sexes in this part, and throughout the article, were analysed separately. This is because males and females differ in body size and sex-specific social preferences [54]. We normalize the eight measures in each dataset to equal variance and zero mean and obtain factor loadings for each sex. Data from the 5.5 to 10 min mark of selection trials were used to obtain the reference factors. Before this time point, most behavioural measures were highly variable over time. The data from the 1 min to 5.5 min mark were saved for later investigation of temporal effects. For each sex, we used the Varimax method to find *m* orthogonal factors. We performed Horn's parallel analysis using R v. 3.1.2 to determine the number of factors *m*. A three-factor model resulted for the females, while a two-factor model was sufficient for males. To test the robustness of these factors, we also ran the same analysis on data from the other experiments, and for combined datasets, with consistent results (see the electronic supplementary material). The factor loadings for the selection experiment data are presented in table 2, while the correlation matrices are shown in the electronic supplementary material. None of the factors identified were significantly correlated with body size (which we obtained from the tracking software) (table 3).

**Table 2.** Factor loadings from the selection experiment. Listed values have final absolute loadings above 0.4. Shown beneath each factor name is the percentage of variance between individuals that is explained.

	females (*N* = 232)			males (*N* = 224)	
	activity	sociability	factor 3	activity	sociability
	(27%)	(20%)	(18%)	(33%)	(19%)
speed	0.61		0.77	0.82	
turning rate	−0.99			−0.81	
dist. from centre		−0.55			
speed corr.	0.40		0.50	0.47	0.41
reaction time					
n.n. align.	0.69	0.41	0.49	0.90	
n.n. dist.		−0.63			−0.69
group size		0.75		0.46	0.71

**Table 3.** Summary of effect sizes on the factor scores, where the latter are calculated using the factor loadings in table 2.

	females			males		
effect	activity	sociability	*N*	activity	sociability	*N*
body size	−0.1	1.1	216	−0.7	−0.2	208
trial segment	−2.6*	−2.4*	58	−5.0***	2.8**	56
predation	−2.5*	2.1*	78	0.0	3.9***	51
sorting rank	6.3***	4.1***	562			
sorting round	−2.7**	2.6**	562			
sorting round * rank	−3.2**	−0.1	562			

The first two effects were tested using the selection experiment: body size is the size in pixels obtained from tracking, and the trial segment is categorical, with the second half of the trial as the high level. The next two were tested using the predation experiment: the high level is a high-predation stream. The final two effects were measured from the sorting experiment: sorting rank increases for higher global alignment, and sorting round is the round number ranging from 1 to 12. All effect sizes are *t*-statistics from linear mixed-effect models. Significance is indicated by *(*p* < 0.05), **(*p* < 0.01) and ***(*p* < 0.001). *N* indicates the number of samples (individuals for body size, missing for four trials; 2 × number of groups for trial segment; number of groups for all others).

For both males and females, the most dominant factor can be identified as collective activity (table 2). This appears to be similar to activity or exploration measures commonly assayed in individual animals [55,56] but may include a socially facilitated component [57]. With this in mind, we will use ‘activity’ as a shorthand. Guppies scoring higher in activity are faster and have lower turning rates. We find that this factor is also associated with higher cross-correlations in speed, probably due to more intense speed bursting during forward movement. More active guppies are also more aligned with their nearest neighbours. The same set of measures map onto this factor (absolute loading greater than or equal to 0.4) between males and females and in the same directions, apart from an extra loading of group size in the males. By comparing factor scores to those obtained for the first half of the trials (controlling for replicate as a random effect), we find that activity decreased over the course of the trials for both sexes (females *t* = − 2.6, *p* = 0.01, males *t* = − 5.0, *p* < 0.001).

The second factor for both males and females reflects a tendency to stay together in a group (table 2). Small nearest neighbour distances and larger group sizes are the strongest loadings on this factor (note that nearest neighbour distances do not load on to factor 1 for either sex). This factor was identified in seven of the eight datasets, including combined datasets. In six of these, nearest neighbour alignment was also found as a positive loading. This factor is similar to a trait that is often tested experimentally in an individual fish by looking at a fish's time spent with conspecifics on the other side of a transparent barrier [58–61] or by latency in joining a group [38]. This trait is usually labelled sociability, and we thus adopt this name hereafter. Sociability decreased for the second half of the trial in females and increased in males (females *t* = − 2.4, *p* = 0.02, males *t* = 2.8, *p* = 0.007). In the selection experiment, this factor is also associated with lower distances from the centre of the arena in females, and higher speed correlations in males; however, these loadings were not found in other datasets (see the electronic supplementary material). The third factor identified in females was not consistent across datasets, and hence we do not interpret it (see the electronic supplementary material).

**Figure 1.**
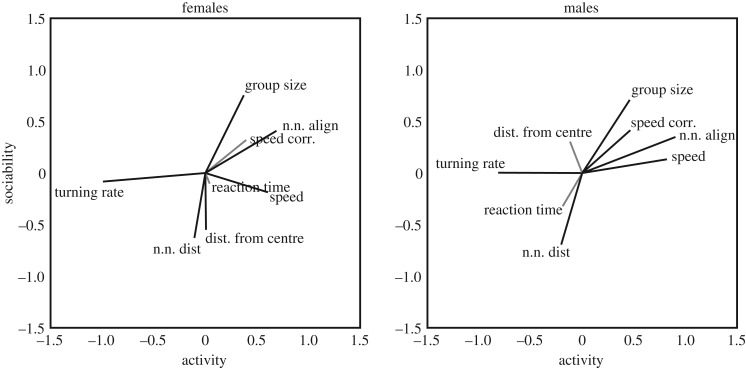
Factor loadings for the activity and sociability factors, plotted as vectors. To read this plot, begin from the centre of the plot and choose one particular measure. As you move outwards along this measure's vector, this measure increases, and affects the factors of sociability and activity in the respective vertical and horizontal displacements. For example, in females, as the turning rate increases, this decreases activity, but has little effect on sociability. Darker vectors indicate measures with at least one factor loading above 0.4.

The respective loadings for the sociability and activity factors are shown in figure 1, revealing how each of our eight collective motion measurements contribute to these factors. Activity and sociability provide an overall model for guppy shoaling behaviour. The activity and sociability factors allow us to see that traditional independent measures of collective motion, i.e. attraction and alignment [24,62], are intertwined. Although sociability and activity are orthogonal to each other, they both contain nearest neighbour alignment for most datasets. Therefore with respect to alignment, sociability and activity are not completely independent, i.e. increased alignment contributes to increased activity and increased sociability. The relationship between activity and alignment has been observed in other fish species, with faster moving fish aligning more [19,32,35,37]. This relationship also arises naturally from models of collective motion [35]. However, the ratio of the nearest neighbour alignment loading onto activity/sociability is 0.69/0.41 = 1.68 for females and 0.90/0.35 = 2.57 for males, indicating that alignment is somewhat more important in the activity factor than it is in the sociability factor. Other measures are more tied to specific factors. The turning rate, for example, decreases the activity factor but does not influence the sociability factor. Indeed, goal-orientated behaviour is classically associated with a reduction in turning rates and more purposeful movements [49].

## 4. Sociability and activity under predation

We then tested how groups varied in terms of sociability and activity when originating from different environments. In this experiment, we collected guppies from four high-predation sites and four low-predation sites. We call this the *predation experiment*. High-predation sites contain either the major predator of adult guppies (*Crenicichla frenata*) or other predatory fish species (*Hoplias malabaricus*, *Aequidens pulcher*), whereas these predators do not occur at low-predation sites [63].

We calculated the eight collective measures for each fish in the predation experiment and calculated the means for each trial, this time using data from the 2nd to the 10th min. This is due to the longer acclimation period in this set-up, and hence the fish requiring a shorter time to start actively exploring the arena. We then used the factor loadings calculated from the selection experiment to obtain factor scores for each trial. Differences in the group factor scores between high-predation and low-predation populations were then tested using a linear mixed-effect model, including river as a random effect, and mean body size as a fixed effect.

**Figure 2.**
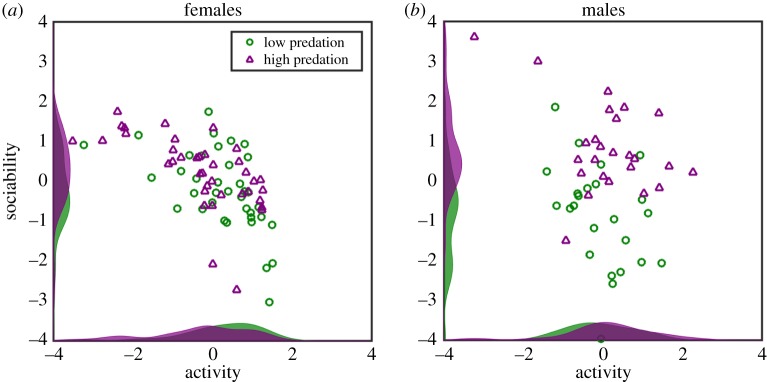
Groups in the Trinidad predation experiment plotted by the activity and sociability factors obtained in §3 using data from the 2nd to 10th min of trials, for (*a*) females and (*b*) males. Along each axis are kernel-smoothed distributions (Gaussian, bandwidth 0.4) of the data points for that factor. (Online version in colour.)

Groups of eight females from high-predation streams scored lower in the activity factor (*t* = − 2.5, *p* = 0.01) and higher in the sociability factor (*t* = 2.1, *p* = 0.04) compared to female groups from low-predation streams. The third female factor was positively correlated with the average body size of the group in this experiment (*t* = 2.6, *p* = 0.01). A strong predation effect was also found in male sociability (*t* = 3.9, *p* < 0.001), with high-predation males more sociable than low-predation males, while no predation effect was found for activity (*t* = 0.02, *p* = 0.99). The activity and sociability factor scores per group are shown in figure 2.

## 5. Effect of sorting for group alignment

As a final test of how effective the factor analysis is in capturing differences between the collective properties of groups, we performed the analysis on a third dataset; *the sorting experiment*. In the sorting experiment, we tested whether groups that were sorted according to the group's average directional alignment differed in terms of their sociability and activity measures [45]. For each of three replicate lines (i.e. independent sorting experiments using different fish), we started with 16 groups of eight female guppies (we did not perform experiments with male fish). The sorting procedure proceeded as follows: in every round of sorting (12 sorting rounds in total in each replicate line), the 16 groups of eight fish were ranked according to their average directional alignment within a circular arena, calculated as
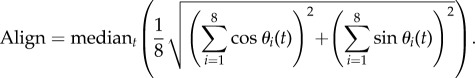


Pairs of adjacently ranked groups were then mixed prior to the next round of assays. For example, four individuals from the top-ranked group (group 1) were mixed with four members of the second-ranked group (group 2), and the other four members of the top-ranked group were mixed with the other four members of the second-ranked group. This procedure was repeated for the third- and fourth-ranked groups, and so on for all pairs of groups in a single round of sorting. Although we sorted for the groups’ average directional alignment, because we take the global measure of alignment (i.e. including all individuals in the arena), this measure could be affected by both sociability (tendency of individuals to aggregate) and activity (how fast and straight individuals were moving) [45].

We have shown previously that over the twelve rounds of sorting, the group rankings according to alignment became more stable, indicating that the individuals were becoming sorted according to behavioural traits. We then showed that the higher-ranked groups displayed higher speed and stronger alignment and aggregation responses, according to various measures [45]. Here, as for the other datasets, we quantify these differences in terms of the activity and sociability axes identified above. We calculated the mean of the eight collective measures for fish in each trial from the 5.5 to 10 min mark. Again, we used the factor loadings calculated from the selection experiment to obtain factor scores for each trial. We then used a linear mixed-effect model with replicate line (*n* = 3) as a random effect, and round number, group ranking and their interaction as fixed effects. We then used these models to assess how the activity and sociability factors changed over the course of the sorting procedure (i.e. over the 12 rounds of sorting) and according to the rank of the groups.

**Figure 3.**
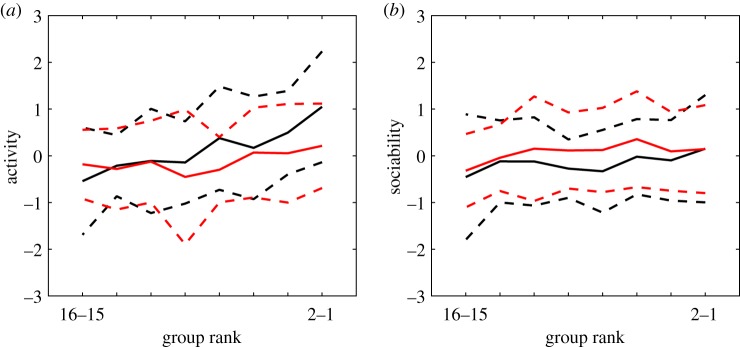
Factor scores for (*a*) activity and (*b*) sociability at the beginning and end of the sorting experiment. The mean (solid lines) ± 1 s.d. (dotted lines) of group factors are plotted against ranking according to alignment. Data are corrected for variation between replicates, and grouped by the first four rounds (black lines) and the final four rounds (red lines). (Online version in colour.)

Figure 3 shows the mean and standard deviation in factor scores calculated from the first four and final four rounds, plotted against group ranking. The activity factor increased with higher group ranking (*t* = 6.3, *p* < 0.001) and decreased over successive rounds (*t* = − 2.7, *p* = 0.006), with a negative interaction term (*t* = − 3.2, *p* = 0.001). This indicates that groups with higher average directional alignment were more active, but this effect decreased as the fish were assayed in successive rounds of sorting. The sociability factor also increased with higher ranking (*t* = 4.1, *p* < 0.001), with more sociable groups having higher degrees of average alignment. This stands in contrast to the results from the predation experiment where, in females, activity and sociability were negatively associated with one another. This highlights that the factor analysis can uncouple activity and sociability across different datasets, albeit with the same species. Notably, the sociability factor also increased with round number (*t* = 2.6, *p* = 0.009). As this is in the same direction as the group rank effect, it could be argued that the top-ranked groups simply habituated faster to the arena. However, if this same argument is applied to activity, it would mean that the top-ranked groups habituated slower. Therefore, the differences between the sorted groups cannot be explained solely by different rates of habituation to the experimental arena.

## 6. Conclusion

We have identified two key components of guppy collective behaviour: (i) sociability, which is associated with aggregation, and (ii) activity, which is associated with coordinated movement. Our three observational and experimental studies show that these components can be separated or coupled, but do not always scale in the same direction. In female guppies under increased predation pressure, fish had increased sociability and decreased activity. In male guppies under increased predation pressure, fish had increased sociability, but consistent levels of activity. When we sorted individuals for higher alignment in the sorting experiment, both sociability and collective activity increased. Table 3 summarizes these results. Both of these behavioural axes are consistent with repeatable temperaments previously found in guppies [58–60].

Other recent work has explained the collective properties of animal groups by breaking down the behaviour of individuals into distinct behavioural axes [37]. Jolles *et al.* [37] tested individual fish and found that they showed consistent inter-individual variation in ‘boldness/exploration’ and ‘sociability’, with sociability scores being negatively correlated with the speed of a fish. Moreover, the authors found that exploration and sociability axes were not correlated with each other. When the authors placed individuals into groups together, the average boldness and sociability scores of group members could explain within-group spatial assortment, inter-group structural differences and the groups' performance in foraging tasks [37]. Our study provides support for the notion that the behaviour of individuals in groups can be broken down into distinct behavioural axes. Indeed, the notion that sociability is an important factor shaping individuals' behaviour in groups is consistent between the two studies. On the other hand, our results suggest that in guppies, speed is more associated with activity (or exploration) than sociability, and sociability and activity can be positively correlated in some systems (i.e. the sorting experiment) and uncoupled, or negatively correlated in others (i.e. the predation experiment). Therefore, beyond our work on guppies, viewing collective motion on two distinct axes of sociability and activity could potentially be a useful general framework for disentangling many of the various and inter-related properties of moving groups. For example, free-swimming sticklebacks tend to be less tightly aggregated when travelling at higher speeds, suggesting that sociability and activity are negatively correlated for this species [37,64]. On the other hand, faster travelling schools of Pacific blue-eyes were more densely aggregated than slower moving groups [35], suggesting a positive relationship between activity and sociability in this species. Using activity and sociability to classify the group-level properties of animal groups could prove a more rigorous way to identify differences in the structure and behaviour of groups compared to more classical attempts (e.g. ‘shoaling’ or ‘schooling’ fish). Of course, this is currently speculation, and further factor analysis on a wider range of populations and species is necessary to confirm this.

An advantage of the method we have applied here is that multiple measurements of collective motion (speed, nearest neighbour distance, etc.) can be combined and interpreted in biologically meaningful ways. As the measures are normalized during the factor analysis, we expect that experimental differences, such as using a larger arena, would cause only a minor change to the pattern of covariances among our chosen measurements. This is supported by the factor analysis from the predation data (see the electronic supplementary material), which used an arena of different size and shape but led to very similar factors. Thus, given any set of reasonable behavioural measurements of a particular species, we expect that a component reflecting activity and one reflecting sociability could be determined. Instead of insisting that behaviour correspond to predefined terms (attraction/alignment) from simulation models, sociability and activity could be as a useful general way of categorizing both between individual differences and between population differences. One potential general measure for sociability could come from studies of group size distributions, where—despite enormous variation between physiology of species, between environments and between experimental set-ups—a single parameter related to the rate at which groups merge and split can be used to compare very different species' tendencies to aggregate [65,66].

We started this article by discussing two distinct evolutionary explanations of collective behaviour: information transfer and the dilution effect. Previous modelling studies associated alignment responses with transfer of information, and attraction responses with dilution [12,22,27,28]. The question now is whether we can associate the collective activity and sociability components, which we have empirically established from movement data, with the evolutionary explanations of information transfer and dilution?

We think we can. Sociability is primarily related to explanations based on dilution effects, because it characterizes the tendency to aggregate, to stay in a group and have more neighbours. As predation pressure on guppies acted most strongly on sociability (this factor was different in both male and female datasets between high- and low-predation populations), we would suggest that the primary evolutionary pressure acting on guppies in high-predation environments is to stay together, rather than to share information. This may be due to the type of predation imposed on guppies. Many of their predators attack in short bursts, striking from ambush locations with sustained chases being uncommon [67,68]. As long as larger groups do not encounter these predators disproportionally more than smaller groups, belonging to a larger group and being closer together can reduce individual risk through simple dilution effects. We might expect differences in sociability to evolve within populations in a similar manner as it does for individual traits, such as boldness [69]. For example, Jolles *et al.* [37] found that in sticklebacks, less sociable individuals were then more likely to be found towards the front of groups, guide group movement and were more likely to discover food first in a foraging context. More relatively sociable individuals, however, showed less variation in the amount of food they consumed, highlighting a trade-off between sociability and the reliability of food intake rates [37]. Within selfish-herd groups, sociability may well be part of a wider behavioural strategy that is similar to being cautious and waiting for other individuals to take actions and locate resources.

We would further suggest that the activity axis is primarily associated with exchange of directional information. A similar relationship between near-neighbour alignment and speed correlations has been observed in a wide range of fish species, and is associated with both milling and directional motion [19,32,35]. These types of collective motion are in turn associated with species which live in more open, less spatially complex environments, such as the open ocean, where attacks from predators can be sustained for periods of hours [70–73]. In another context, Pettit *et al.* found that larger, faster pigeons were more likely to lead flocks than smaller, slower birds [74]. Here the transfer of information is with regard to navigation, but is also transmitted by speed correlation and alignment responses.

In conclusion, activity and sociability appear to occur on orthogonal axes of behavioural variation. Using the factor analysis, it seems possible to determine whether activity and/or sociability are important in a given species' collective behaviour. Moreover, it is also possible to determine how these factors differ between different populations or selection lines. Investigating these two axes may shed significant insight into the evolution of collective behaviour in other animal groups.

## Supplementary Material

Supplementary Information

## Data accessibility

Data deposited in http://10.6084/m9.figshare.5165854.
