# Supplementary material for: Using activity and sociability to characterize collective motion
Source: Philos Trans R Soc Lond B Biol Sci. 2018 Mar 26;373(1746):20170015. doi: 10.1098/rstb.2017.0015 (PMC5882985; doi:10.1098/rstb.2017.0015)
Supplement: Supplementary Information [file rstb20170015supp1.pdf]

Using activity and sociability to characterise collective motion: Supplementary Information

December 1, 2017

A Correlation matrices

	S_i	T_i	D_i	C_i	R_i	A_i	N_i	G_i
speed S_i	1.000	-0.544	0.134	0.568	-0.186	0.717	-0.225	0.367
turning rate T_i	-0.544	1.000	0.041	-0.393	-0.049	-0.691	0.138	-0.412
dist. from centre D_i	0.134	0.041	1.000	-0.212	-0.038	-0.201	0.181	-0.441
speed corr. C_i:	0.568	-0.393	-0.212	1.000	-0.207	0.621	-0.458	0.577
reaction time R_i	-0.186	-0.049	-0.038	-0.207	1.000	-0.151	0.261	-0.156
n.n. align A_i	0.717	-0.691	-0.201	0.621	-0.151	1.000	-0.538	0.740
n.n. dist. N_i	-0.225	0.138	0.181	-0.458	0.261	-0.538	1.000	-0.636
group size G_i	0.367	-0.412	-0.441	0.577	-0.156	0.740	-0.636	1.000

Table 1: Correlation matrix for female data in the selection experiment.

	S_i	T_i	D_i	C_i	R_i	A_i	N_i	G_i
speed S_i	1.000	-0.662	-0.130	0.482	-0.267	0.787	-0.277	0.453
turning rate T_i	-0.662	1.000	0.060	-0.351	0.128	-0.738	0.162	-0.374
dist. from centre D_i	-0.130	0.060	1.000	0.033	-0.069	0.009	-0.214	0.147
speed corr. C_i:	0.482	-0.351	0.033	1.000	-0.217	0.559	-0.356	0.536
reaction time R_i	-0.267	0.128	-0.069	-0.217	1.000	-0.277	0.322	-0.285
n.n. align A_i	0.787	-0.738	0.009	0.559	-0.277	1.000	-0.425	0.666
n.n. dist. N_i	-0.277	0.162	-0.214	-0.356	0.322	-0.425	1.000	-0.581
group size G_i	0.453	-0.374	0.147	0.536	-0.285	0.666	-0.581	1.000

Table 2: Correlation matrix for male data in the selection experiment.

B Factor analysis: Robustness

When data was combined, the individual datasets were first centred and normalised. Larger datasets were sampled at random to equal the size of the smallest dataset. Factor tables on the left are computed using all eight measures, while tables on the right are computed without the reaction time measure. For each table, factors are ordered by the amount of variation explained.

Females: Selection (N=232)			
	Activity (27%)	Sociability (20%)	Factor 3 (18%)
speed	0.61		0.77
turning rate	-0.99		
dist. from centre		-0.55	
speed corr.	0.40		0.50
reaction time:			
n.n. align:	0.69	0.41	0.49
n.n. dist.:		-0.63	
group size:		0.75	

No reaction time		
	Activity (36%)	Sociability (24%)
speed	0.83	
turning rate	-0.72	
dist. from centre		-0.56
speed corr.	0.54	
n.n. align:	0.86	0.44
n.n. dist.:		-0.61
group size:	0.44	0.83

Females: Predation (N=624)			
	Activity (31%)	Factor 2 (13%)	Factor 3 (7%)
speed	0.84		
turning rate	-0.93		
dist. from centre			
speed corr.			
reaction time:		-0.61	
n.n. align:	0.95		
n.n. dist.:			-0.55
group size:		0.66	

No reaction time			
	Activity (35%)	Factor 2 (16%)	Factor 3 (11%)
speed	0.86		
turning rate	-0.92		
dist. from centre			
speed corr.			
n.n. align:	0.93		
n.n. dist.:		0.99	
group size:			0.79

Females: Sorting round 1 (N=384)		
	Sociability (26%)	Activity (22%)
speed		0.71
turning rate		-0.67
dist. from centre		
speed corr.	0.42	
reaction time:		
n.n. align:	0.58	0.81
n.n. dist.:	-0.95	
group size:	0.71	

No reaction time		
	Sociability (29%)	Activity (26%)
speed		0.72
turning rate		-0.66
dist. from centre		
speed corr.	0.42	
n.n. align:	0.56	0.83
n.n. dist.:	-0.95	
group size:	0.71	

Females: Combined (N=696)		
	Activity (28%)	Sociability (18%)
speed	0.79	
turning rate	-0.79	
dist. from centre		
speed corr.		
reaction time:		
n.n. align:	0.90	0.42
n.n. dist.:		-0.78
group size:		0.65

No reaction time		
	Activity (29%)	Sociability (24%)
speed	0.75	
turning rate	-0.77	
dist. from centre		
speed corr.		0.45
n.n. align:	0.85	0.52
n.n. dist.:		-0.87
group size:		0.65

Males: Selection (N=224)		
	Activity (33%)	Sociability (19%)
speed	0.82	
turning rate	-0.81	
dist. from centre		
speed corr.	0.47	0.41
reaction time:		
n.n. align:	0.90	
n.n. dist.:		-0.69
group size:	0.46	0.71

No reaction time		
	Activity (39%)	Sociability (18%)
speed	0.83	
turning rate	-0.81	
dist. from centre		
speed corr.	0.49	
n.n. align:	0.92	
n.n. dist.:		-0.65
group size:	0.49	0.71

Males: Predation (N=408)		
	Sociability (24%)	Activity (23%)
speed		0.72
turning rate		-0.95
dist. from centre		
speed corr.		
reaction time:		
n.n. align:	0.67	0.56
n.n. dist.:	-0.59	
group size:	0.85	

No reaction time		
	Sociability (26%)	Activity (25%)
speed		0.73
turning rate		-0.94
dist. from centre		
speed corr.		
n.n. align:	0.69	0.55
n.n. dist.:	-0.60	
group size:	0.85	

Males: Combined (N=448)		
	Sociability (26%)	Activity (22%)
speed		0.74
turning rate		-0.81
dist. from centre		
speed corr.	0.44	
reaction time:		
n.n. align:	0.63	0.66
n.n. dist.:	-0.59	
group size:	0.91	

No reaction time		
	Sociability (27%)	Activity (25%)
speed		0.75
turning rate		-0.83
dist. from centre		
speed corr.	0.42	
n.n. align:	0.64	0.64
n.n. dist.:	-0.61	
group size:	0.86	

All data (N=896)		
	Activity (25%)	Sociability (21%)
speed	0.77	
turning rate	-0.82	
dist. from centre		
speed corr.		
reaction time:		
n.n. align:	0.78	0.53
n.n. dist.:		-0.69
group size:		0.76

No reaction time		
	Activity (29%)	Sociability (23%)
speed	0.78	
turning rate	-0.79	
dist. from centre		
speed corr.		
n.n. align:	0.78	0.55
n.n. dist.:		-0.72
group size:		0.75
